# Three-gene PCR and high-resolution melting analysis for differentiating vertebrate species mitochondrial DNA for biodiversity research and complementing forensic surveillance

**DOI:** 10.1038/s41598-020-61600-3

**Published:** 2020-03-16

**Authors:** Daniel O. Ouso, Moses Y. Otiende, Maamun M. Jeneby, Joseph W. Oundo, Joel L. Bargul, Scott E. Miller, Lillian Wambua, Jandouwe Villinger

**Affiliations:** 1 International Centre of Insect Physiology and Ecology (icipe), P.O. Box 30772–00100, Nairobi, Kenya; 2 Biochemistry Department, Jomo Kenyatta University of Agriculture and Technology (JKUAT), P.O. Box 62000–00200, Nairobi, Kenya; 3 Kenya Wildlife Service, Veterinary Department, P.O. Box 40241-00100, Nairobi, Kenya; 4 Institute of Primate Research, National Museums of Kenya, Department of Tropical and Infectious Diseases, P. O. Box 24481-00502, Karen, Nairobi, Kenya; 5 National Museum of Natural History, Smithsonian Institution, Washington, DC USA; 6 Present Address: International Livestock Research Institute, Department of Animal Biosciences, P.O. Box 30709-00100, Nairobi, Kenya

**Keywords:** High-throughput screening, Biodiversity, Genetic variation, Genetic markers

## Abstract

Reliable molecular identification of vertebrate species from morphologically unidentifiable tissue is critical for the prosecution of illegally-traded wildlife products, conservation-based biodiversity research, and identification of blood-meal hosts of hematophagous invertebrates. However, forensic identification of vertebrate tissue relies on sequencing of the mitochondrial cytochrome oxidase I (*COI*) ‘barcode’ gene, which remains costly for purposes of screening large numbers of unknown samples during routine surveillance. Here, we adapted a rapid, low-cost approach to differentiate 10 domestic and 24 wildlife species that are common in the East African illegal wildlife products trade based on their unique high-resolution melting profiles from *COI*, cytochrome b, and *16S* ribosomal RNA gene PCR products. Using the approach, we identified (i) giraffe among covertly sampled meat from Kenyan butcheries, and (ii) forest elephant mitochondrial sequences among savannah elephant reference samples. This approach is being adopted for high-throughput pre-screening of potential bushmeat samples in East African forensic science pipelines.

## Introduction

Unsustainable hunting, consumption, and sale of bushmeat in Africa contribute immensely to the decline of threatened wild animal species. The global bushmeat trade is valued at several billion US dollars. Up to 270 tons of bushmeat were flown into Europe through a single airport in 2010 from Africa^1^. While this is a major crisis for wildlife in central and western Africa, it is a growing concern in eastern and southern Africa^2^. Efforts to regulate or prevent illegal wildlife trade depends on accurate, efficient, and sustainable tools for species identification of confiscated and surveillance samples.

Illegal wildlife trade is mainly fuelled by the need for diet and income supplementation^3^. The consequences of direct human contact with bushmeat have been severe. A classic example of disease originating from or harboured by bushmeat is Ebola virus disease, which has infected humans upon contact with infected wild animals such as fruit bats, nonhuman primates, and forest antelopes^4,5^. The impact of bushmeat hunting on animal populations can also be severe^6^. Many favoured wild animal species for bushmeat are already endangered, some close to extinction^7^. There are also flow-on effects to the ecosystems^8^ and tourism.

Concerted efforts have been put in place to save precious flora and fauna, including awareness campaigns and fencing of parks and conservancies. However, cases of bushmeat hunting are still rampant even with laws prohibiting bushmeat trade. Law enforcement can only be effective when backed by efficient prosecution, which relies on proper surveillance, concrete evidence, and informed policies. Accurate identification of suspect samples forms the basis of forensic evidence, which currently relies widely on sequencing of the barcode cytochrome c oxidase subunit I (*COI*) gene^9,10^, the gold-standard for vertebrate species determination implemented by initiatives such as the International Barcode of Life project (iBOL) (www.ibol.org)^11,12^. DNA barcoding enables fast, reliable, cost-effective, and automatable species determination by users with limited taxonomic experience^10^ and is increasingly becoming accepted and adopted as a means of court-admissible evidence generation for wildlife crime prosecutions in Africa^13–17^. However, as only a small proportion of potential samples sequenced are of illegally traded wildlife products, the cost of surveillance by mass-barcode sequencing is high and thus not sustainable in the long term.

While emerging technologies, such as MinION sequencing (Oxford Nanopore Technologies, Oxford, UK), can facilitate rapid on-site DNA metabarcoding of environmental samples^18^, costs of library preparation and adaptors required to associate specific sequences with individual samples remain high and are not amenable to on-going screening of smaller sample sizes compared to standard PCR. Continuous and rapid vertebrate species identification amidst scarce resource availability (i.e. no in-house sequencing capacity) requires techniques that are rapid and have limited post-PCR processing. Restriction fragment length polymorphism^19^, random amplification of polymorphic DNA^20^, and amplified fragment length polymorphism^21^ have been used for vertebrate species identification, but still require post-PCR processing and are limited to detecting very specific wildlife species and/or suffer from poor reproducibility, and thereby limit the development of reference databases^22^.

To address this lack of rapid and cost-effective techniques for robustly identifying vertebrate species from morphologically indistinct samples, we adapted and validated a real-time high-resolution melting (HRM) analysis-based approach, which requires no post-PCR processing, to rapidly identify and differentiate domestic species from wild vertebrate species commonly targeted for bushmeat in East Africa^23^. HRM analysis is a fast, sensitive, and specific tool developed for genotyping PCR product sequence variations^24^ that employs the use of intercalating fluorescent dyes, such as EvaGreen^25^, which undergo rapid solvent fluorescence quenching as duplex-DNA PCR products are slowly melted within an HRM-capable real-time PCR thermocycler. The amplicon melting temperatures (T_m_) and specific melt curve shapes are dependent on DNA complementarity, the order of DNA bases, G-C content, and amplicon length. PCR-HRM has been used with a number of genes to identify species among diverse viruses^26^, bacteria^27^, malaria *Plasmodium*^28^, mosquitoes^29^ and their bloodmeals^30^, plant products^31^, and animals within discrete families^32,33^, as well as human individuals^34^. It is quick (occurs within a real-time PCR machine immediately after PCR) and does not require downstream analyses such as gel electrophoresis to determine amplification success or PCR-product sequencing to differentiate sequence variants.

Relatively, HRM analysis provides an efficient and sustainable species identification workflow, especially because the number of samples in a run can vary from one sample to over 90 samples without significant differences in per-sample analysis costs. However, HRM analysis has not yet been standardized to support forensic pipelines for identifying illegally traded wildlife products. By systematically comparing HRM profiles generated by *COI*, cytochrome b oxidase (*cyt b*), and *16S* ribosomal (r)RNA gene PCR products, we show that this approach can robustly differentiate domestic vertebrate species from wildlife species and can be used to triage forensic barcode sequencing confirmations such that they are limited to only wildlife specimens. During the validation process, we made interesting observations on the mitochondrial history of East African elephants. We further used the approach in a proof-of-concept study to identify illegal bushmeat among samples covertly purchased from butcheries in the Naivasha region of Kenya.

## Results

### Species-specific mitochondrial gene HRM analysis profiles

**Figure 1:**
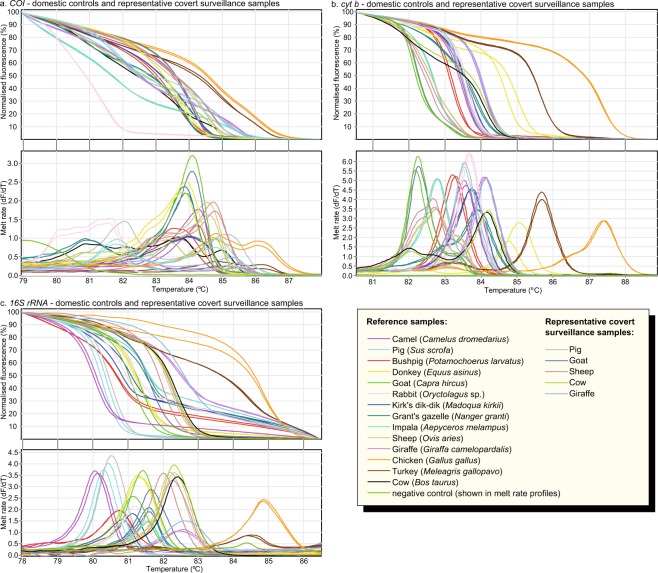
Distinct normalised HRM and melt rate profiles of domestic reference and representative covert surveillance samples. Normalised HRM profiles are represented as percent fluorescence and melt rates are represented as change in fluorescence units with increasing temperatures (dF/dT) for (**a**) *COI*, (**b**) *cyt b*, and (**c**) *16S rRNA* markers.

**Figure 2:**
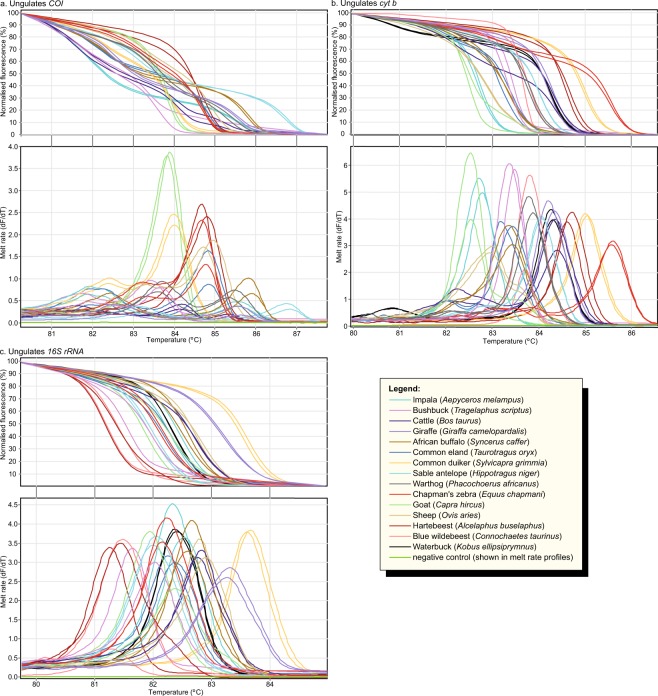
Distinct PCR-HRM profiles of ungulate species. Normalised HRM profiles are represented as percent fluorescence and melt rates are represented as change in fluorescence units with increasing temperatures (dF/dT) for (**a**) *COI*, (**b**) *cyt b*, and (**c**) *16S rRNA* markers.

**Figure 3:**
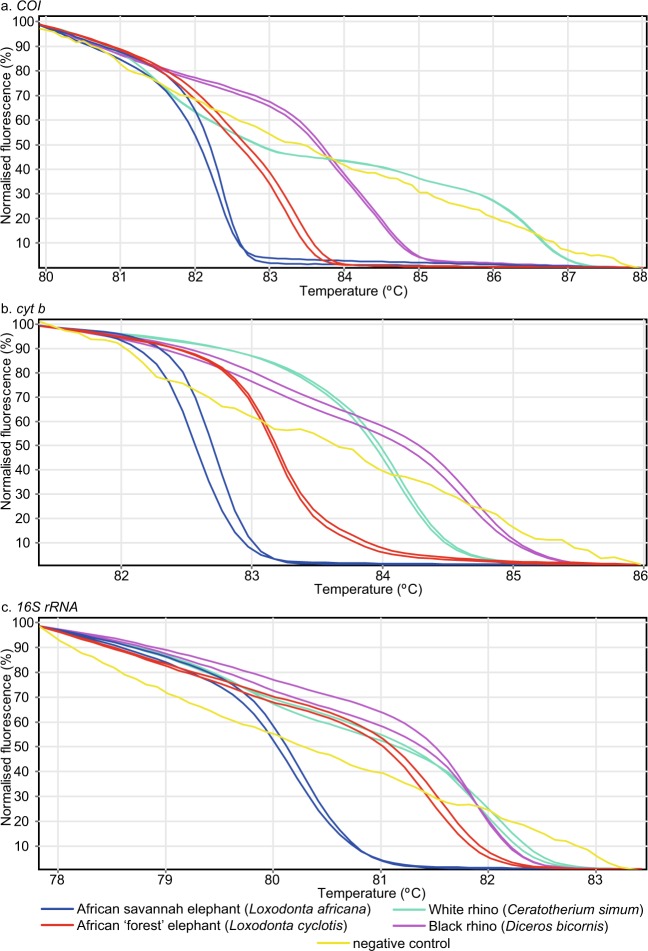
Distinct PCR-HRM profiles among elephant and rhino species. Normalised HRM profiles are represented as percent fluorescence for (**a**) *COI*, (**b**) *cyt b*, and (**c**) *16S rRNA* markers. The African forest elephant mitochondrial amplicons were obtained from African savannah elephant reference samples.

**Figure 4:**
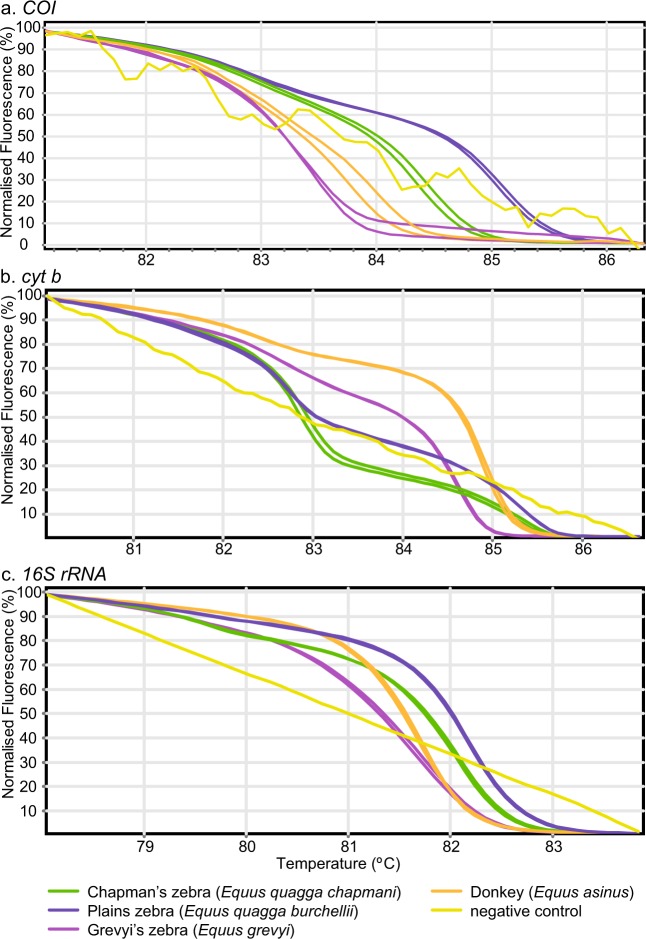
Distinct PCR-HRM profiles for the Equidae family showing the differentiation of two zebra species (one with two sub-species) and donkey. Normalised HRM profiles are represented as percent fluorescence for (**a**) *COI*, (**b**) *cyt b*, and (**c**) *16S rRNA* markers.

**Figure 5:**
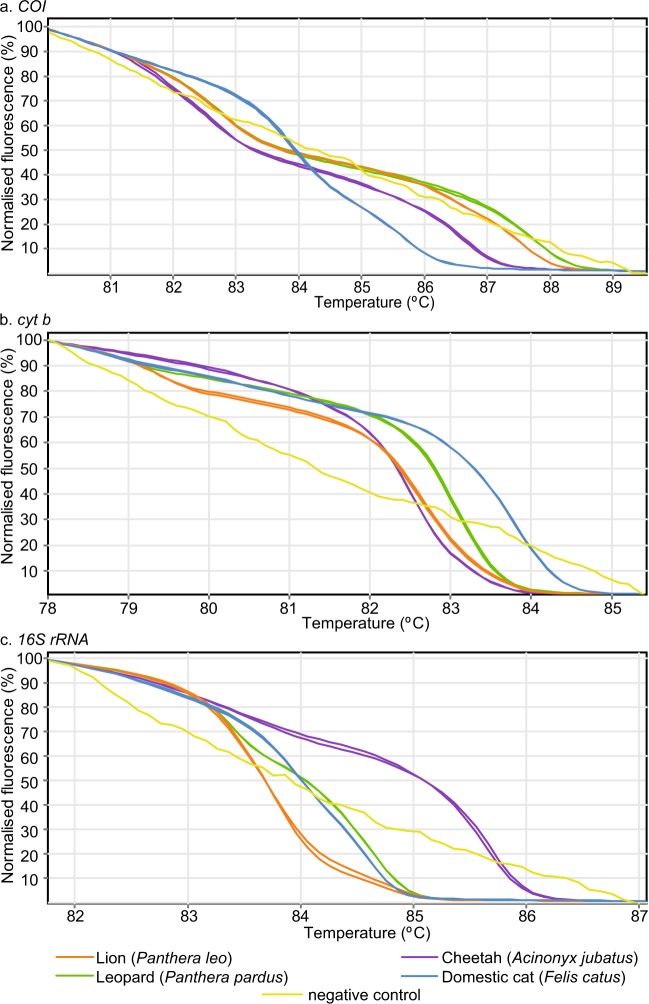
Distinct PCR-HRM profiles for Felidae family species. Normalised HRM profiles are represented as percent fluorescence for (**a**) *COI*, (**b**) *cyt b*, and (**c**) *16S rRNA* markers.

We generated HRM profiles from short mitochondrial *COI*, *cyt b*, and *16S rRNA* PCR products to differentially identify the species of 107 reference tissue samples (Supp. Table 1), which included 10 domestic and 24 East African wildlife vertebrate species or sub-species (18 Bovidae, four Equidae, four Felidae, two Elephantidae, two Rhinocerotidae, three Suidae, and one Camelidae species) (Figs. 1, 2, 3, 4, 5). Where available, we used multiple samples for particular species. Species identifications were further confirmed by sequencing of the *COI* barcode region. The domestic species, including three members of the Bovidae family (cattle-*Bos taurus*, goat-*Capra hircus*, and sheep-*Ovis aries*), one member each for Suidae (pig-*Sus scrofa*), Equidae (donkey-*Equus asinus*), Camelidae (camel-*Camelus dromedarius*), and Leporidae (rabbit-*Oryctolagus* sp.), and two Phasianidae (turkey-*Meleagris gallopavo* and chicken-*Gallus gallus*), were successfully differentiated from all other tested wild animal specimens by three-marker PCR-HRM analysis.

**Figure 6:**
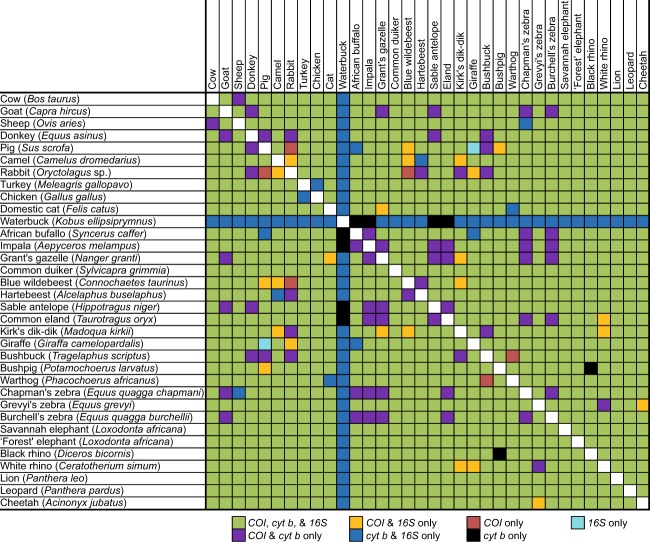
Summary matrix of pair-wise discriminations by PCR-HRM of 34 species and DNA marker resolution. Markers that generated distinct HRM profiles for pair-wise species comparisons are indicated by colours according to the legend.

Though most species could be differentiated by pairwise comparisons of HRM profiles using all three markers (473 pairs), some species pairs were only distinguishable by certain markers, or a combination of markers (Fig. 6), due to similar HRM profiles within 1 °C melting temperature (T_m_) ranges or poor or non-amplification of some species with the primers of particular markers. For example, waterbuck (*Kobus ellipsiprymnus*) failed to amplify with *COI*, but was differentiated from all other species based on its *cyt b* and *16S rRNA* HRM profiles. Some species showed similarities in both shapes and melting temperature for particular markers. For example, among *COI* HRM profiles, pig samples generated similar *COI* and *cyt b* HRM profiles within a 1 °C T_m_ range to those generated by giraffe (*Giraffa camelopardalis*) samples, but could be clearly differentiated based on their distinct *16S rRNA* HRM profiles (Figs. 2 and 6). Specific pair-wise species differentiations that could be made by HRM analysis of each of the three markers are shown in Fig. 6.

We generated two distinct sets of HRM profiles for elephant reference samples obtained from Kenya Wildlife Service (KWS) using all three markers (Fig. 3). Upon *COI* barcode sequencing of these samples, we found that one set of HRM profiles corresponded to the expected savannah elephant (*Loxodonta africana*), which is native to Kenya (*COI* GenBank accession MN124271; *cyt b* GenBank accession MN124234). Interestingly, the other set of HRM profiles were generated from savannah elephants with forest elephant (*Loxodonta cyclotis*) mitochondrial DNA (mtDNA) (*COI* GenBank accession MN124272; *cyt b* GenBank accession MN124235), not native in Kenya. All markers were also able to distinguish the two species of rhinos, black (*Diceros bicornis*) and white (*Ceratotherium simus*) rhinos (Fig. 3). Among equine samples, zebra species and sub-species native to East Africa (Burchell’s plains zebra-*Equus quagga burchellii*, Chapman’s plains zebra-*Equus quagga chapmani*, Grévy’s zebra-*Equus grevyi*) and donkey (Fig. 4), as well as available Felidae reference samples (cheetah-*Acinonyx jubatus*, leopard***-****Panthera pardus*, lion-*Panthera leo*, domestic cat-*Felis catus*) (Fig. 5), were clearly distinguished by three-marker PCR-HRM from each other and from other domestic and wildlife species (Figs. 1–3, 6). During early HRM experiments using DNA extracts provided by KWS, we also obtained unique PCR-HRM profiles for loggerhead sea turtle (*Caretta caretta*) and green sea turtle (*Chelonia mydas*) (Supp. Figure 1).

### Marker discrimination comparison

We did not encounter any species among those tested that could not be distinguished from others by the combined analysis of HRM profiles generated by all the three mtDNA markers. To compare species detection power of molecular markers, the resolution of *COI, cyt b*, and *16S rRNA* PCR-HRMs were compared. Based on 561 pair-wise comparisons of species differentiation (Fig. 6); 39 pairs (7%) could not be distinguished by *COI* PCR-HRM, of which 33 pairs were due to non-amplification of a species (waterbuck) during PCR, and 12 (2.3%) and 33 (6.3%) pairs could not be distinguished by *cyt b* and *16 rRNA* PCR-HRM, respectively. Although PCR-HRM analysis of the *COI* marker was consistently best at resolving species for DNA samples that amplified, giving unique melt curve profiles in shape and peak T_m_, the *cyt b* and *16S rRNA* markers had better PCR efficiency in all cases for any particular sample, observed by the lower C_T_ values and higher fluorescence values in the melt curve plot. The *cyt b* marker resolved species better than the *16S* marker, which had the highest number of species pairs with similar PCR-HRM profiles. While it is expected that longer PCR products would have more than one melt peak due to the tendency to have multiple melting domains, *cyt b* (~383 bp) and *16S rRNA* (~200 bp) PCR products tended to have simple single-peaks compared to *COI* PCR products (~205 bp), for which many samples had multiple peaks, generating a greater diversity of unique HRM profiles.

### Comparison of DNA extraction protocols

The commercially available Qiagen DNeasy Blood and Tissue Kit is used widely across molecular biology laboratories. In comparing this kit with a cheaper laboratory-optimised SDS-proteinase K protocol, we mostly observed similar HRM curve profiles, however with T_m_ shifts ranging from −0.02 to + 0.4 °C, + 0.15 to +0.25 °C and +0.13 to +0.35 °C for *COI, cyt b* and *16S rRNA* markers, respectively, when using the laboratory-optimised protocol compared to the Qiagen protocol (Supp. Figure 2).

### Blind validation

To validate the approach, we blindly identified 49 DNA extracts, whose species origin were only known to KWS colleagues who supplied the samples. Through the combined PCR-HRM analysis of the three markers, we were able to correctly identify 48 (98%) of the samples, with at least two markers (Supp. Table 2). However, due to T_m_ shifts resulting from different extraction protocols used among supplied extracts that had been extracted at different time points, four (8%) of these samples were correctly identified, but with relatively low confidence. One impala extract was incorrectly identified (with low confidence) as common eland. We were not able to rule out sample contamination as some of the DNA extracts had arrived with opened tubes.

### Proof-of-concept HRM identification of covertly sampled meat from rural Kenyan butcheries

We covertly sampled 90 meat samples with support of the KWS from butcheries in the Naivasha region of Kenya (0 °43′ 0.01′′ N 36° 26′ 9.28′′ E, about 77 km from the capital Nairobi). Naivasha is near wild animal conservancies and game parks. We identified one sample that was sold as legal domestic meat (from Kambi Samaki area) to be giraffe bushmeat by PCR-HRM and subsequent *COI* barcode sequencing confirmation (GenBank accession MN124280). The remaining 89 samples consisted of 49 (54.4%) sheep, 29 (32.2%) cattle, eight (8.9%) goats, and two (2.2%) pigs, while one (1.1%) sample failed to amplify. Out of the 17 random samples whose species identity were given by the butcher at the point of sale, six samples (35.3%) sold as goat meat were confirmed by PCR-HRM analysis and *COI* barcode sequencing to be sheep meat (GenBank accession MN124281).

## Discussion

This study clearly demonstrates the utility of PCR-HRM analysis of three mtDNA markers for efficiently differentiating and identifying the vertebrate species origin of unknown tissue samples. Using PCR-HRM, we were able to differentiate domestic and wild animal species native to East Africa by HRM analysis of short *COI*, *cyt b*, and *16S rRNA* gene PCR amplicons. Further, we used the approach to blindly identify illegal giraffe bushmeat among meat samples purchased from rural and urban butcheries, using forensic barcode sequencing only for confirmation purposes. Therefore, the PCR-HRM approach presented here represents a valuable complement to molecular forensic pipelines for the surveillance of illegal wildlife products as it eliminates the need for mass barcode sequencing of suspect specimens, most of which tend to be legally traded domestic animal samples. These assays can also be effectively used for biodiversity surveys from the blood-meals of hematophagous invertebrates^35^ and for consumer protection purposes to ensure that meat products for consumption are labelled properly.

We analysed the PCR-HRM profiles generated by the three DNA markers from 10 domestic and 24 wildlife species native to East Africa, demonstrating the capacity of this approach to differentiate a large range of species. While we were able to differentiate all the species when considering the combined analysis of HRM profiles generated by all three mtDNA markers, the HRM profiles of individual markers could not be used to distinguish particular vertebrate species pairs, which may be attributed to the varied resolution strengths of the three markers^36,37^. Though some studies have only considered one^38^ or two^39^ different DNA markers, this study demonstrates increased robustness of species identification by PCR-HRM when using combined analysis of three mtDNA markers. Though reliable and widely applied as the gold standard in species identification, the high costs associated with sequencing may not be sustainable in some instances, especially where large sample sizes are analysed^26^ and when sequencing is outsourced. PCR coupled to HRM offers a quicker, cheaper, and relatively easy-to-work-with real-time PCR alternative^24^.

We demonstrated the applicability of PCR-HRM analysis to illegal bushmeat surveillance with a small-scale covert surveillance exercise conducted in collaboration with the KWS. Using the three PCR-HRM assays to screen 90 meat samples sold as domestic livestock meat in Naivasha, Kenya, we only had to sequence eight representative DNA samples with unique HRM profiles to confirm species identifications. This translated into a 91% reduction in sequencing costs (~US$750 savings after subtracting costs of two extra PCR reactions for 90 samples) compared to direct sequencing of all the 90 samples. Among the eight samples sequenced, one was confirmed to be from giraffe, as identified by HRM analysis. The other seven were representative sequences of samples with HRM profiles matching those of domestic livestock species. In addition to the giraffe bushmeat, we identified sheep meat that was sold by local butcheries as goat meat. This further demonstrates the potential utility of PCR-HRM for surveillance by consumer protection agencies, such as the Kenya Bureau of Standards (KEBS), which in turn, could inform policy formulation and law enforcement.

Finding a giraffe sample among only 90 specimens was surprising, as we expected poaching of much smaller, easier to trap, ruminants to occur more frequently among illegal bushmeat^23^. Poached giraffe meat products are of particular concern as giraffe populations have been declining in the region. The latest update of the International Union of Conservation of Nature (IUCN), *Red List2018-2*, recently added two of the nine sub-species of giraffes to the “Critically Endangered” category. Five out of seven assessed giraffe sub-species are categorised between “Near Threatened” to “Critically Endangered” (IUCN *Red List2018-2*). Further, the sale of illegal bushmeat as livestock meat presents a public health concern as unsuspecting consumers may be exposed to a higher risk of contracting zoonotic diseases^4,5^ by unknowingly consuming bushmeat.

The ability to distinguish sub-species of plains zebra, suggests that the three-marker PCR-HRM method can differentiate not only closely related ungulates, but also some sub-species. We also note that the *cyt b* and *16S rRNA* HRM profiles for cow, goat, sheep, pig, and chicken samples obtained in this study are comparable to those previously obtained from mosquito blood-meals analyses to determine host feeding preferences^30^, despite differences in tissue type and PCR cycling conditions. These observations support the overall reproducibility of the method. Nonetheless, we observed melt temperature frame shift generally resulting in slightly higher T_m_ across species when DNA samples were extracted using a laboratory-optimised SDS-proteinase K DNA extraction procedure rather than the commercial Qiagen DNeasy Blood and Tissue Kit, likely due to differences in salt concentrations within the DNA elutes of the different extraction techniques^40^. The larger shift seen in *COI*, compared to the others, might be attributed to the thermodynamic effects to its also unique multi-modal melting domains. Therefore, for comparable and reproducible HRM results, all samples should be processed under the same conditions. However, with more affordable (and probably advanced) HRM-capable thermocyclers entering the market, such as the MIC-4 (Bio Molecular Systems, Australia) and Chai’s Open qPCR (Chai, CA, USA) thermocyclers, cross-platform comparisons may be aided by novel algorithms to harmonize HRM analysis across platforms^41^.

The 98% identification success rate demonstrated by blind validation further exemplifies the power of the assay. However, among the samples that were identified, four (8%) had similar shapes to reference controls, but with T_m_ shifts on some markers. These T_m_ shifts can be attributed to the different extraction protocols used at KWS before receiving extracts as observed in our controlled comparisons of results arising from extracts using different protocols, despite our attempts to normalise for this by re-precipitation of extracts prior to PCR-HRM analysis. PCR-HRM analysis of an impala sample extract (#69) identified the sample incorrectly as common eland. As some of the tubes unfortunately arrived in the laboratory with opened lids, we suspect that this misidentification may be due to cross-contamination of DNA during transport, which must be avoided for reliable molecular analysis of samples.

During the validation of several elephant reference samples, we identified two sets of distinct HRM profiles among the KWS stock samples of Kenyan savannah elephants (Fig. 3, Supp. Table 2). We determined, through *COI* barcode sequencing, that some samples amplified mtDNA sequences associated with forest elephant populations, which are thought not to exist in the region^42^. The forest elephant mitochondria amplicons had distinct HRM profiles using all three markers from samples with savannah elephant mitochondria. This finding could represent an artefact of past hybridization between female forest elephants and male savannah elephants^43^, or male forest elephants and female savannah elephants with paternal mtDNA transfer, as recently observed in humans^44^, after which the forest elephant mtDNA persisted in East African savannah elephant populations. Alternatively, these findings may be due to unknown past translocations of elephants from the range of the forest elephants. Though we cannot explain these findings conclusively, our findings demonstrate that the method can also be used to identify mtDNA variants within populations. Additional screening of savannah elephant samples by PCR-HRM could determine the frequency of forest elephant mtDNA in savannah elephant populations and potentially *vice versa*, possibly unravelling important hybridization or range expansion histories.

The differences exhibited by the mtDNA markers in their ability to differentiate any two species by PCR-HRM strongly support the complementarity in using the combination of the three optimised mtDNA markers, which addresses marker-specific shortcomings in differentiating certain vertebrate species. Previous studies also highlighted the importance of marker complementarity in screening mosquitoes for blood-meal sources using HRM^30^. Even though this means that two to three PCR assays must be run to confidently identify a species, the overall time and cost is still cheaper than mass-barcode sequencing, as the runs can be done simultaneously. Moreover, there was still no need for large-scale sequencing of all PCR amplicons. Only a few representatives with unique peaks need to be selected for species confirmation through DNA sequencing.

We found that the ~200-bp *16S rRNA* amplicons had lower HRM resolving power than the *COI* (~205 bp) and *cyt b* (~383 bp) amplicons. The use of short gene targets, which are more suitable for HRM analysis, for barcode identification of species has been shown to be almost as effective as longer barcode sequencing targets^45^ and are more suitable for environmental samples. The *16S rRNA* amplicon region might have been too short to incorporate sufficient sequence variations required to distinguish species as effectively as the other two markers. However, previously, only a marginal positive correlation between amplicon length and species resolution based on *COI* sequence amplicons was identified^46^. Nonetheless, our findings are consistent with a study that found *16S rRNA* sequences to be 2.5 times less variable than *COI* and *cyt b* sequences among rodents within the tribe Praomyini^45^. In contrast to previous studies that have investigated the use of HRM analysis to differentiate vertebrate species using different primers to target discrete taxonomic groups^32^, our study used three different sets of universal primers to “globally” differentiate a large repertoire of species. This suggests that its applicability could be much broader than previously published assays.

## Conclusions

The methodology presented here provides an efficient and reliable workflow for differentiating and identifying vertebrate species of DNA samples without having to sequence the majority of sample PCR products. For research and forensic scenarios involving vertebrate species not represented in this study or with limited available control reference samples, dominant species (e.g. livestock) can be sequenced and used as controls to triage all other species with non-matching HRM profiles for barcode sequencing. The need for positive controls within laboratories may be reduced with increased implementation and data-sharing to generate on-line reference databases of HRM profiles from different real-time PCR platforms and laboratories, and machine learning algorithms that can be used to develop automated HRM classifications^26,41^.

Despite the challenges in our proof-of-concept exercise in which we covertly sampled meat from butcheries, during times not favouring the concealed nature of illegal bushmeat trade (from late morning to early evening) and having to mitigate the alerting-appearance of the KWS covert operations team and vehicle, we used the three-marker PCR-HRM methodology to identify one bushmeat specimen among the butchery-sampled meat. This shows that the approach can effectively identify illegal bushmeat trade products.

## Methods

### Samples for optimisation

Samples including muscle, blood, and hide were provided by the Kenya Wildlife Service (KWS) forensic laboratory. Some of the domestic species were purchased from local supermarkets or butcheries and were confirmed by long COI barcode gene sequencing. We targeted, among others, commonly hunted bushmeat species, and common domestic species (cattle, goat, sheep, donkey, pig, camel, rabbit, turkey, chicken, and cat). KWS meat samples exhibited varying levels of integrity depending on their state at the point of confiscation. Some of the samples were older than five years. After confiscation or sampling, meat and blood had been stored in −40 °C freezers in the KWS forensic laboratory. Hide had been stored at room temperature. Samples were grouped mainly according to their taxonomic families, including Bovidae, Equidae, Felidae, Hominidae, Elephantidae, Rhinocerotidae, and Suidae. The numbers of species within each taxonomic family included in the study was limited by the availability of samples (Supp. Table 1).

### Genomic DNA extraction from meat and blood tissue

We extracted genomic DNA from meat and blood using Qiagen’s DNeasy Blood and Tissue Kit (Qiagen, Hannover, Germany) according to the manufacturer’s instructions, with minimal modifications as follows; 25 μl of proteinase K was used for 2-hour incubations of muscle and hard tissue or 1-hour incubations of blood samples. For comparison purposes, we also extracted total DNA from meat and blood using a laboratory-optimised protocol, with minor variations based on sample types (meat, blood). Briefly, in 1.5-ml microcentrifuge tubes, about 3 mm^3^ of meat was added to 450 μl of lysis buffer (10 mM Tris (pH 8.1), 0.5% SDS, 5 mM EDTA and 200 μg/ml proteinase K) or 70 μl of blood was topped up to 450 μl with the lysis buffer. Meat samples were then incubated at 65 °C in a water bath for 2 hours, whereas blood samples were incubated for one hour. This was followed by the addition of 150 μl of protein precipitation solution (8 M Ammonium acetate, 1 mM EDTA) at room temperature, then incubation in ice for 15 minutes. The resulting precipitates were centrifuged for 15 minutes at 25000 relative centrifugal force (rcf) in a 5417 R Eppendorf centrifuge at 4 °C (Eppendorf, Hamburg, Germany). The resultant supernatants were equilibrated to room temperature and then carefully drawn and added into fresh 1.5 ml tubes containing 400 ml of isopropanol; the pellets were discarded. The mixtures were inverted 100 times followed by centrifugation at room temperature for 15 minutes at 25000 rcf. The resulting supernatants were discarded and 800 μl of 70% cold ethanol was added to each pellet, then inverted several times before centrifugation at 25000 rcf for 15 minutes at 4 °C. Subsequently, excess ethanol was carefully decanted off and then pellets were inverted over paper towels to air-dry. DNA was resuspended in PCR grade water and stored at −20 °C until use. Replicate extractions were performed on samples of species for which available verified specimens were limited to one (see Supp. Table 1).

### Polymerase chain reaction

In separate single-plex PCRs, we used primers targeting vertebrate *COI* (Uni-MinibarF1: 5′-TCCACTAATCACAARGATATTGGTAC-3′; Ronping_R: 5′-TATCAGGGGCTCCGATTAT-3′)^35,46^, *cyt b* (Cyt b For: 5′-CCCCTCAGAATGATATTTGTCCTCA-3′; Cyt b Rev: 5′-CCATCCAACATCTCAGCATGATGAAA-3′)^47^, and *16S rRNA* (Vert *16S* For: 5′-GAGAAGACCCTRTGGARCTT-3′; Vert *16S* Rev: 5′-CGCTGTTATCCCTAGGGTA-3′)^31^ gene fragments of ~205 bp, ~383 bp, and ~200 bp, respectively. Ten-microlitre PCRs contained 2 μl of pre-formulated 5X HOT FIREPol^®^ EvaGreen^®^ HRM Mix, no ROX (Solis BioDyne, Tartu, Estonia), forward and reverse primers (Macrogen, Europe) at a final reaction concentration of 0.5 µM and 2 μl of DNA template. PCRs were performed in an HRM-capable Rotor-Gene Q thermocycler (Qiagen, Hilden, Germany). Every run had a set of known positive control samples as well as a no-template negative control. The amplification cycling conditions included an initial hold at 95 °C for 15 minutes, then 40–45 cycles (for raw curve fluorescence plateauing) of denaturation at 95 °C for 20 seconds, annealing at 56 °C for 20 seconds and extension at 72 °C for 30 seconds, followed by a final extension at 72 °C for 5 minutes. All three markers were optimised for the same cycling conditions. Fluorescence was acquired on the green channel with a 470-nm emission and 510-nm detection spectrum.

### High-resolution melting analysis

Immediately after PCR amplification, HRM ensued; amplicons were gradually melted at 0.1 °C increments from 75 °C to 95 °C, with fluorescence acquisition every 2 seconds. From the fluorescence acquisition data, raw graphs of fluorescence against temperature (°C) were automatically generated by the Rotor-Gene Q Series Software. We selected HRM and melt curve analyses from the analysis tab on the Rotor-Gene Q Series Software (2.3.1 build 49). The raw HRM graphs were normalised for fluorescence range of between zero and 100 using the ‘Normalisation Region 1' and ‘Normalisation Region 2' parameters, with widths of 0.1 °C. Both the leading and trailing normalization ranges had varying start temperatures depending on the marker and the species-profile-spread within the graph in a run. They were used to remove fluorescence “noise” (the non-sigmoid ends) from the ends of the HRM profiles. The melt curve analysis transformed the HRM profiles into bell-shaped profiles with peaks, enabling peak differentiation and providing an alternate data visualisation. Both the HRM and melt-curve graphs were used to infer species.

Unknown melting profiles were compared to the profiles of a set of determined controls on a per-target basis. The profile comparisons were based on melt-curve peaks and curve shapes. Samples with similar profiles (peak and shape) within 1 °C across all (and at least two) amplified markers were considered to be the same species. We made identifications systematically, first identifying species with similar *COI* HRM-curve shapes within 1 °C, then narrowing down these species based on those with similar *cyt b* profiles within 1 °C, and finally correlating these with the *16S rRNA* profiles. To confirm HRM-based species identifications relative to positive controls, we sequenced PCR-HRM products. For *cyt b* and *16S rRNA,* we directly purified amplicons using ExoSAP-IT (USB Corporation, Cleveland, OH) according to the manufacturer’s protocol, then sent for Sanger sequencing at Macrogen, Europe. Since the PCR-HRM target for *COI* lies within the longer barcoding *COI* region, we sequenced the full *COI* barcode region rather than the *COI* HRM products.

### Long COI barcode sequencing

We used conventional PCR for long *COI* barcode sequencing in 15-μl reaction volumes including 3 μl of 5X HOT FIREPol^®^ Blend Master Mix (Solis BioDyne, Tartu, Estonia), forward and reverse primers at 0.5 µM final concentrations, and 2 μl of a template. We used previously described primers VF1d (TCTCAACCAACCACAARGAYATYGG) and VR1d (TAGACTTCTGGGTGGCCRAARAAYCA)^48^ tagged with M13 tails TGTAAAACGACGGCCAGT and CAGGAAACAGCTATGAC, respectively, as adapters for vertebrate barcode sequencing based on the long (750 bp) *COI* gene. Amplification conditions included an initial hold at 95 °C for 15 minutes, then 40 cycles of denaturation at 95 °C for 20 seconds, annealing at 57 °C for 30 seconds and extension at 72 °C for 60 seconds, followed by a final extension at 72 °C for 7 minutes. We electrophoresed PCR products for 45 minutes at 100 V in 2% agarose gels in 1X TAE buffer to ensure proper amplification of target sequences. We then purified amplicons with clear bands using ExoSAP-IT (USB Corporation, Cleveland, OH), according to manufacturer protocol and sent them for Sanger sequencing at Macrogen, Europe.

### Sequence analysis

All sequences were trimmed, edited and analysed using Geneious v10.2.6 (available from http://www.geneious.com) software^49^ created by Biomatters and queried in GenBank using default BLAST^50^ parameters and aligned sequences obtained with corresponding GenBank reference sequences. We confirmed of the identities obtained by HRM analysis through sequencing of the *cyt b, 16S rRNA*, and long *COI* fragments and MAFFT sequence alignments with identical GenBank reference sequences (Nucleotide nr collection) retrieved using Geneious software’s megablast program. We used the default Geneious settings for all other parameters. The *COI* and *cyt b* sequences obtained from reference samples were deposited into GenBank (*COI* accessions MN124245-MN124279; *cyt b* accessions MN124208-MN124240, MN124243-MN124244) and are shown in Supp. Table 1.

### Blind validation

For blind validation, KWS provided 49 DNA samples that were known to KWS (Supp. Table 2) but not to the researchers conducting HRM analysis, as well as labelled positive control DNA from green sea turtle, blue wildebeest, common eland, hippopotamus, bushpig, impala, donkey, dik-dik, common duiker, African savannah elephant, African ‘forest’ elephant, warthog, sable antelope, cattle, goat, sheep, waterbuck, zebra, Grant’s gazelle, and pig. As the unknown samples were extracted using various methods, we normalised for potential extraction differences by re-precipitating the DNA extracts. Briefly, 20 μl of DNA (some, that were evaporated, were topped up with PCR-grade water) was added to fresh 1.5-ml microcentrifuge tubes, followed by the addition of 10 μl of 5 M ammonium acetate and then 60 μl of cold 100% ethanol. After vortexing briefly for 10 seconds, the mixtures were kept on ice for 30 minutes and then centrifuged at full speed for 30 minutes in a cooled fixed angle Eppendorf centrifuge (5417 R). The supernatants were then carefully decanted off and the pellets were air dried by inverting over paper towels. The DNA was resuspended in 20 μl PCR-grade water and stored in −20 °C until PCR-HRM analysis.

### Proof-of-concept HRM analysis of covertly sampled meat from rural Kenyan butcheries

Open butcheries were covertly sampled in 22 demarcations within Naivasha (N0°43′ 0.01′′ E36° 26′ 9.28′′ E), between 10 am and 4 pm. They included Kambi Daraja, Gilgil, Kinamba, Kasarani, Kihoto, Kamere, Kwa Muya, Kongoni, DCK, Ndabib, Duro, Kabati, Kanjoo Estate, Mirema, Sanctuary, Karagita, Langalanga, Kikopey, Kambi Somali, Kongasis, Mutaita, and Hell’s Gate. Longonot (S00°55.096′ E036°31.407′) and Mai Mahiu (S00°58.966′ E036°35.103′) were sampled along the way, between Naivasha and Nairobi. Samples were separately wrapped and stored inside the vehicle in a cooler box with dry ice for sub-sampling later the same day. We sub-sampled each sample in triplicate into 1.8-ml cryovials using a sterile scalpel and fresh gloves for every sample. Incisions were made on the samples to access the unexposed inner flesh. The samples were immediately stored in liquid nitrogen shipper tanks for transporting to the laboratory and in −80 °C freezers until extraction. In the laboratory, we identified the unknown samples by comparing their *COI, cyt b*, and *16S rRNA* PCR-HRM profiles (melting temperature and curve shape) to those of already known controls and confirmed their identities by amplicon sequencing.

## Supplementary information

Supplementary information.

## Data Availability

The datasets used and/or analysed during the current study are available from the corresponding author on reasonable request. Study sequences have been deposited in to the GenBank nucleotide database (accessions MN124208-MN124240, MN1242043-MN124281).
