# Supplementary material for: Three-gene PCR and high-resolution melting analysis for differentiating vertebrate species mitochondrial DNA for biodiversity research and complementing forensic surveillance
Source: Sci Rep. 2020 Mar 16;10:4741. doi: 10.1038/s41598-020-61600-3 (PMC7075967; doi:10.1038/s41598-020-61600-3)
Supplement: Supplementary file 1 — Supplementary information. [file 41598_2020_61600_MOESM1_ESM.pdf]

Supplementary information

Three-gene PCR and high-resolution melting analysis for differentiating vertebrate species mitochondrial DNA for biodiversity research and complementing forensic surveillance

Daniel O. Ouso, Moses Y. Otiende, Maamun Jeneby, Joseph W. Oundo, Joel L. Bargul, Scott E. Miller, Lillian Wambua, Jandouwe Villinger

Supplementary Table 1. Samples used in the HRM reference optimisations

Animal	Family	Species	Common name	Tissue	Blood	Total	*COI* sequence	*cyt b* sequence
Domestic	Bovidae	*Bos taurus*	Cow	2	4	6	MN124245	MN124208
	Bovidae	*Capra hircus*	Goat	1	1	2	MN124246	MN124209
	Bovidae	*Ovies aries*	Sheep	1	1	2	MN124247	MN124210
	Equidae	*Equus asinus*	Donkey	1	0	1	MN124248	MN124211
	Suidae	*Sus scrofa*	Pig	1	0	1	MN124249	MN124212
	Camelidae	*Camelus dromedarius*	Camel	0	1	1	MN124250	MN124213
	Leporidae	*Oryctolagus cuniculus*	Rabbit	1	0	1	MN124251	MN124214
	Phasianidae	*Meleagris gallopavo*	Turkey	1	0	1	MN124252	MN124215
	Phasianidae	*Gallus gallus*	Chicken	1	1	2	MN124253	MN124216
	Felidae	*Felis catus*	Domestic cat	1	0	1	MN124254	MN124217
Wildlife	Bovidae	*Kobus ellipsiprymnus*	Waterbuck	3	0	3	MN124255	MN124218
	Bovidae	*Syncerus caffer*	African buffalo	4	4	8	MN124256	MN124219
	Bovidae	*Aepyceros melampus*	Impala	4	0	4	MN124257	MN124220
	Bovidae	*Nanger granti*	Grant's gazelle	1	0	1	MN124258	MN124221
	Bovidae	*Sylvicapra grimmia*	Common duiker	3	0	3	MN124259	MN124222
	Bovidae	*Connochaetes taurinus*	Blue wildebeest	5	2	7	MN124260	MN124223
	Bovidae	*Alcelaphus buselaphus*	Hartebeest	1	1	2	MN124261	MN124224
	Bovidae	*Hippotragus niger*	Sable antelope	0	2	2	KY628416*	MN124225
	Bovidae	*Tragelaphus oryx*	Eland	2	0	2	MN124262	MN124226
	Bovidae	*Madoqua kirkii*	Kirk's dik-dik	1	0	1	MN124263	MN124227
	Bovidae	*Tragelaphus scriptus*	Bushbuck	1	2	3	MN124264	MN124228
	Giraffidae	*Giraffa camelopardalis*	Giraffe	4	2	6	MN124265	MN124229
	Suidae	*Potamochoerus porcus*	Bushpig	2	0	2	MN124266	MN124230
	Suidae	*Phacochoerus africanus*	Warthog	2	2	4	MN124267	MN124231
	Equidae	*Equus quagga chapmani*	Chapman's zebra	2	0	2	MN124268	MN124243
	Equidae	*Equus grevyi*	Grevyi's zebra	0	2	2	MN124269	MN124232
	Equidae	*Equus quagga burchellii*	Plain zebra	2	2	4	MN124270	MN124233
	Elephantidae	*Loxodonta africana*	Savannah elephant	2	4	6	MN124271	MN124234
	Elephantidae	*Loxodonta africana*	'Forest' savannah elephant	0	2	2	MN124272	MN124235
	Rhinocerotidae	*Diceros bicornis*	Black rhino	0	4	4	MN124273	MN124236
	Rhinocerotidae	*Ceratotherium simum*	White rhino	0	5	5	MN124274	MN124237
	Felidae	*Acinonyx jubatus*	Cheetah	0	3	3	MN124275	MN124238
	Felidae	*Panthera leo*	Lion	3	3	6	MN124276	MN124244
	Felidae	*Panthera pardus*	Leopard	0	2	2	MN124277	EF056507*
	Cheloniidae	*Chelonia mydas*	Green sea turtle	4	0	4	MN124278	MN124239
	Cheloniidae	*Caretta caretta*	Logger head sea turtle	1	0	1	MN124279	MN124240
			**Total:**	57	50	107		

*Previously published sequences

Supplementary Table 2. Blind identification of DNA extracts

sample #	Blind ID	***COI***	***cyt b***	***16S rRNA***	**Predicted by HRM analysis**	**Vouchered ID**
1	#2	Green sea turtle	Green sea turtle	Green sea turtle	Green sea turtle	Green sea turtle
2	#4	Blue wildebeest	Blue wildebeest	Blue wildebeest	Blue wildebeest	Blue wildebeest
3	#5	Zebra	Zebra	Common Eland, Zebra	Zebra	Zebra
4	#8	Green sea turtle	Poor amplification	Green sea turtle	Green sea turtle	Green sea turtle
5	#11	Zebra	Zebra	Zebra (also common eland, goat)	Zebra	Zebra
6	#12	Common eland	Common eland	Common eland (also cattle)	Common eland	Common eland
7	#13	Green see turtle	Green sea turtle	Green sea turtle	Green sea turtle	Green sea turtle
8	#15	Poor amplification	Green sea turtle	Green sea turtle	Green sea turtle	Green sea turtle
9	#18	Common eland	Common eland	Common eland (also goat, zebra, Grant's gazelle)	Common eland	Common eland
10	#19	Zebra	Zebra	Zebra (also goat)	Zebra	Zebra
11	#24	Green sea turtle	Green sea turtle	Green sea turtle	Green sea turtle	Green sea turtle
12	#25	Zebra	Zebra	Zebra (also goat)	Zebra	Zebra
13	#26	Grant's gazelle	Grant's gazelle	Grant's gazelle (also goat)	Grant's gazelle	Grant's gazelle
14	#27	Grant's gazelle	Grant's gazelle	Grant's gazelle (also goat, zebra)	Grant's gazelle	Grant's gazelle
15	#28	African 'forest' elephant	African 'forest' elephant	African 'forest' elephant	African 'forest' elephant	Elephant
16	#29	African savannah elephant	African savannah elephant	African savannah elephant	African savannah elephant	Elephant
17	#30	Poor amplification	African savannah elephant	African savannah elephant	African savannah elephant	Elephant
18	#34	African 'forest' elephant	African 'forest' elephant	African 'forest' elephant	African 'forest' elephant	Elephant
19	#35	African savannah elephant	African savannah elephant	African savannah elephant	African savannah elephant	Elephant
20	#36	African savannah elephant	African savannah elephant	African savannah elephant	African savannah elephant	Elephant
21	#38	Impala	Impala	Impala	Impala	Impala
22	#39	Impala	Impala	Impala	Impala	Impala
23	#40	Impala	Impala	Impala	Impala	Impala
24	#41	Impala	Impala	Impala	Impala	Impala
25	#43	Grant's gazelle	Grant's gazelle	Grant's gazelle (also goat)	Grant's gazelle	Grant's gazelle
26	#44	Grant's gazelle	Grant's gazelle	Grant's gazelle (also goat)	Grant's gazelle	Grant's gazelle
27	#45	Grant's gazelle	Grant's gazelle	Grant's gazelle (also goat)	Grant's gazelle	Grant's gazelle
28	#48	Dik-dik, Donkey	Dik-dik, common duiker, Sable antelope	Dik-dik	Dik-dik	Dik-dik
29	#49	Grant's gazelle	Grant's gazelle	Grant's gazelle (also goat)	Grant's gazelle	Grant's gazelle
30	#50	Grant's gazelle	Grant's gazelle	Grant's gazelle (also goat)	Grant's gazelle	Grant's gazelle
31	#51	Zebra	Zebra	Zebra (also common eland, goat, impala)	Zebra	Zebra
32	#52	Not Amplified	Donkey	Donkey	Donkey	Donkey
33	#54	Common duiker	Common duiker (also hartebeest, blue wildebeest)	Common duiker (also common eland, cattle)	Common duiker (low confidence)	Common duiker
34	#55	Zebra	Zebra	Zebra (also common eland, goat, impala)	Zebra	Zebra
35	#58	Zebra	Zebra	Zebra (also common eland, goat, impala)	Zebra	Zebra
36	#59	Zebra	Zebra	Zebra (also common eland, goat, impala)	Zebra	Zebra
37	#61	Grant's gazelle	Grant's gazelle	Grant's gazelle (also goat)	Grant's gazelle	Grant's gazelle
38	#62	Grant's gazelle	Grant's gazelle	Grant's gazelle (also goat)	Grant's gazelle	Grant's gazelle
39	#63	Waterbuck (shape variation)	Waterbuck	Waterbuck (with variant secondary peak)	Waterbuck (low confidence)	Waterbuck
40	#64	Common eland	Common eland (in shape, shifted peak)	Dik-dik, pig, common eland (in shape, shifted peak)	Common eland (low confidence)	Common eland
41	#65	Impala	African 'forest' elephant, Impala (in shape, shifted peak)	Impala (also common eland, zebra)	Impala	Impala
42	#66	Dik-dik, donkey	Dik-dik	Dik-dik	Dik-dik	Dik-dik
43	#67	Dik-dik, donkey	Dik-dik	Dik-dik	Dik-dik	Dik-dik
44	#68	Dik-dik, donkey	Dik-dik	Dik-dik	Dik-dik	Dik-dik
45	#69	Common eland	Common eland (far from impala)	Dik-dik, pig, common eland	Common eland (low confidence)	Impala*
46	#70	Dik-dik (shifted peak)	Not amplified	Green sea turtle, dik-dik (shape variation)	Dik-dik (low confidence)	Dik-dik
47	#71	African 'forest' elephant	African 'forest' elephant	African 'forest' elephant	African 'forest' elephant	Elephant
48	#72	African 'forest' elephant	African 'forest' elephant	African 'forest' elephant	African 'forest' elephant	Elephant
49	#73	Common eland, dik-dik, donkey	Common eland	Common eland	Common eland	Common eland

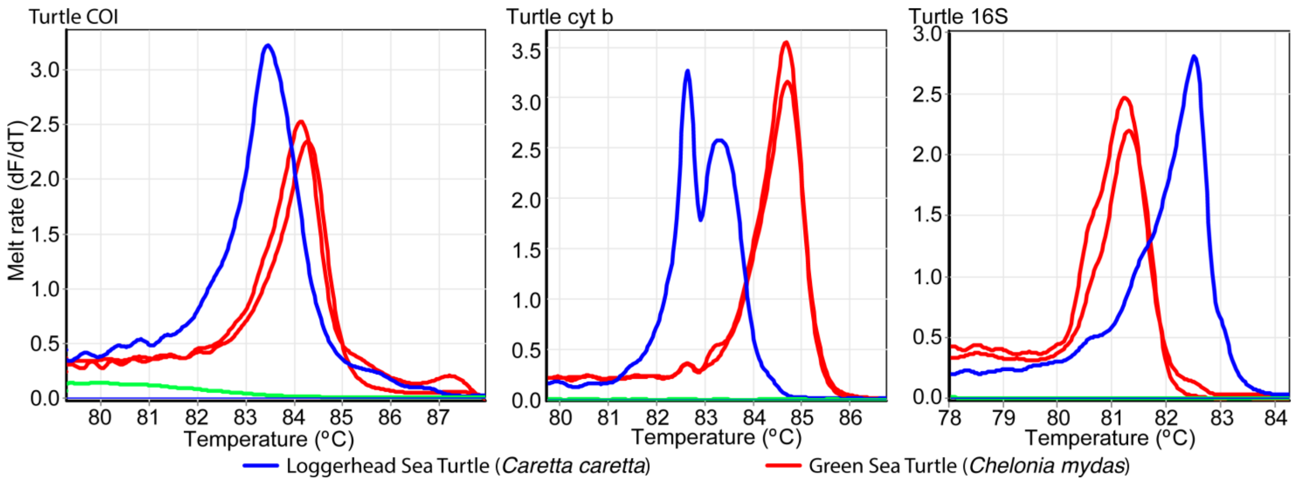

Supplementary Figure 1: Distinct PCR-HRM melt rate profiles for Cheloniidae family species. Melt rate profiles are represented as change in fluorescence units with increasing temperatures (dF/dT) for (a) *COI*, (b) *cyt b*, and (c) *16S rRNA* markers.

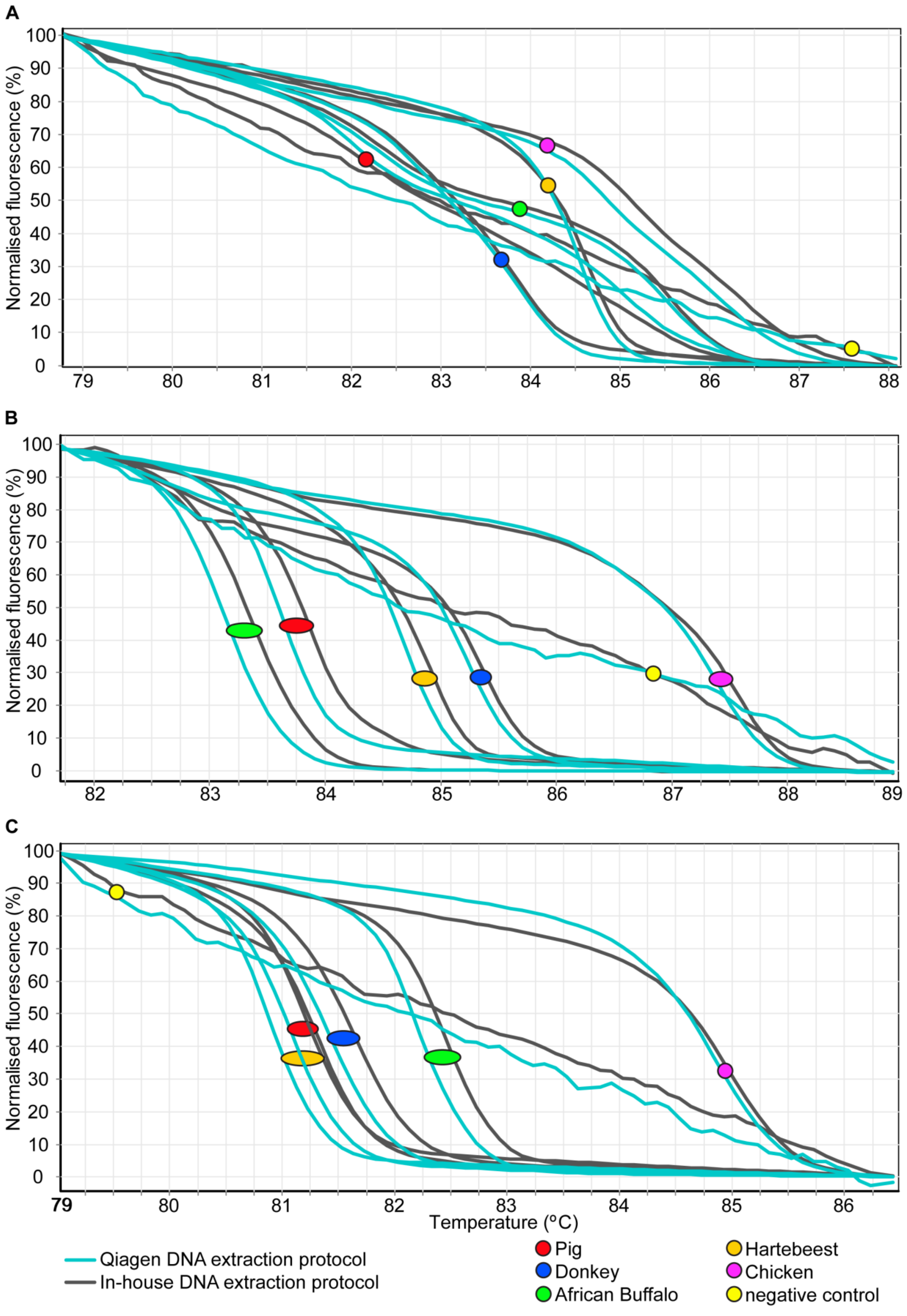

Supplementary Figure 2: Normalised HRM profiles of representative reference samples extracted using different protocols. Normalised HRM profiles are represented as percent fluorescence for (a) *COI*, (b) *cyt b*, and (c) *16S rRNA* markers.
